# Diagnostic Pitfalls in Papillary Muscle Rupture-Associated Acute Mitral Regurgitation after Acute Myocardial Infarction

**DOI:** 10.1155/2021/1396194

**Published:** 2021-12-21

**Authors:** Akiko Kameyama, Hiroshi Imamura, Hiroshi Kamijo, Kanako Takeshige, Katsunori Mochizuki, Kenichi Nitta

**Affiliations:** Department of Emergency and Critical Care Medicine, Shinshu University School of Medicine, 3-1-1 Asahi, Matsumoto, Nagano 390-8621, Japan

## Abstract

Papillary muscle rupture (PMR) is a rare and fatal complication of acute myocardial infarction (AMI). We report a case of acute mitral regurgitation (MR) due to PMR with pulmonary edema and cardiogenic shock following AMI with small myocardial necrosis. An 88-year-old woman was brought to our emergency department in acute respiratory distress, shock, and coma. She had no systolic murmur, and transthoracic echocardiography was inconclusive. Coronary angiography showed obstruction of the posterior descending branch of the right coronary artery. Although the infarction was small, the hemodynamics did not improve. Transesophageal echocardiography established papillary muscle rupture with severe mitral regurgitation 5 days after admission. Thereafter, the patient and her family did not consent to heart surgery, and she eventually died of progressive heart failure. Physicians should be aware of papillary muscle rupture with acute mitral regurgitation following AMI in patients with unstable hemodynamics, no systolic murmur, and no abnormalities revealed on transthoracic echocardiography.

## 1. Introduction

Currently, papillary muscle rupture (PMR) is rare because of improved revascularization techniques; however, it remains as a life-threatening complication of acute myocardial infarction (AMI) and is associated with high mortality [1, 2]. PMR usually occurs 2–7 days after myocardial infarction (MI) and is characterized by acute pulmonary edema and cardiogenic shock [3].

Herein, we report the case of acute mitral regurgitation (MR) due to PMR with pulmonary edema and cardiogenic shock, following AMI with small myocardial necrosis. The patient had no systolic murmur, so MR was not suspected initially. The patient was finally diagnosed with PMR on transesophageal echocardiography (TEE) performed due to the poor quality of images obtained on transthoracic echocardiography (TTE).

## 2. Case Presentation

An 88-year-old woman with diabetes mellitus and hypertension rushed to our emergency department on account of acute respiratory distress, shock, and coma. She had a history of productive cough and intermittent back pain 7 and 4 days before admission, respectively, along with chest pain, which had resolved spontaneously 2 days after its onset. One day prior to admission, she developed acute dyspnea and restlessness. Finally, she became unconscious before being brought to the emergency room.

**Figure 1:**
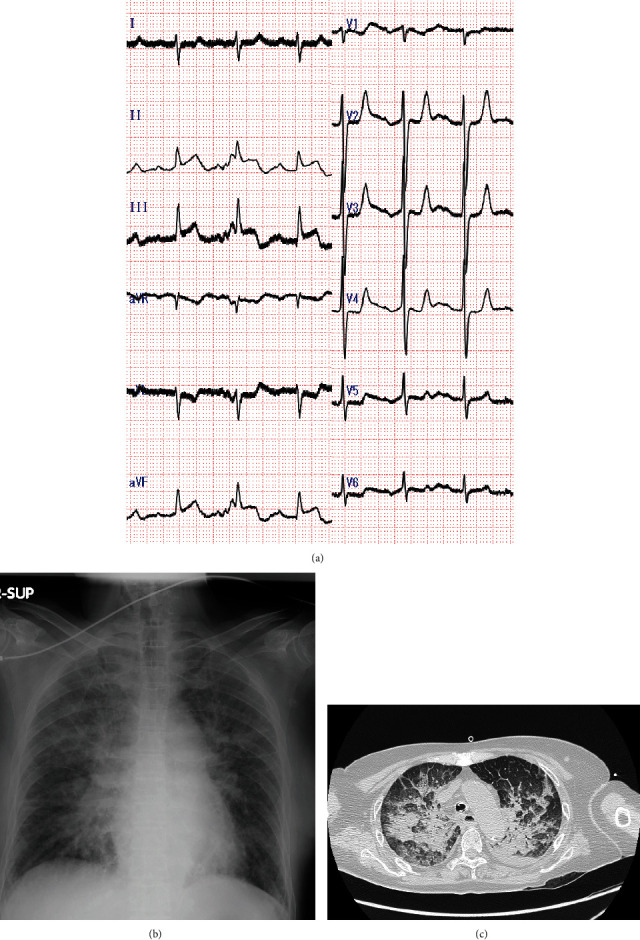
Electrocardiogram, chest radiograph, and computed tomography of the chest on admission. (a) The electrocardiogram on admission showing sinus tachycardia with ST elevation in the leads II, III, and aVF. (b) The chest radiograph on admission showing marked pulmonary congestion. (c) Computed tomography of the chest showing consolidation and ground ground-glass opacity.

**Figure 2:**
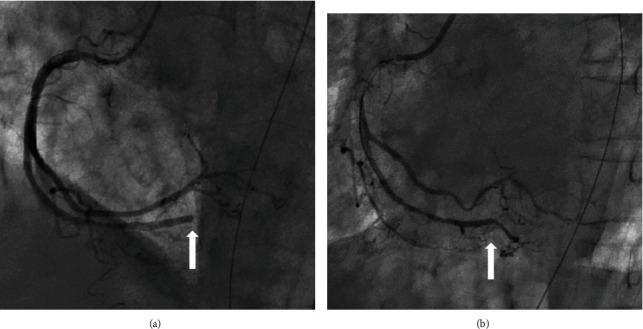
Coronary angiogram. (a) The right coronary artery (RCA) showing a total occlusion of the posterior descending branch (white arrow). (b) Revascularization achieved (white arrow) by performing balloon angioplasty to treat the distal RCA lesion.

On admission, her pulse was palpable and regular with a rate of 90 beats/min; blood pressure was unmeasured. Her respiratory rate could not be ascertained because a bag valve mask was being used; her body temperature was 35.9°C. Bilateral coarse crackles were heard in her chest; no murmurs were heard. She was intubated, and temporarily stabilized; afterward, she regained consciousness. Electrocardiography (ECG) showed sinus rhythm with ST-elevation on leads II, III, and aVF (Figure 1(a)). Chest radiography revealed a butterfly pattern (Figure 1(b)). TTE performed in the emergency room produced poor images, but revealed good left ventricular (LV) function with inferior wall mild hypokinesis and mild-to-moderate MR. Laboratory evaluation revealed the following: white blood cell count, 19200/*μ*L; hemoglobin, 14.3 g/dL; platelet count, 18.4 × 10^4^/*μ*L; aspartate transaminase, 32 IU/L; alanine transaminase, 17 IU/L; lactate dehydrogenase, 416 IU/L; creatinine kinase (CK), 194 IU/L; CK-MB, 9 IU/L; blood urea nitrogen, 18.3 mg/dL; creatinine, 1.71 mg/dL; C-reactive protein (CRP), 9.28 mg/dL; troponin-T, 0.68 mg/dL; D-dimer, 7.8 *μ*g/mL. Computed tomography of the lung revealed consolidation and ground-glass opacity (Figure 1(c)). Emergency coronary angiography showed no significant stenosis in the major coronary arteries, except the total occlusion in the posterior descending branch of the right artery (Figure 2(a)). Balloon angioplasty was performed to restore patency (Figure 2(b)), but the patient remained in shock and oxygenation worsened. An intra-aortic balloon pump (IABP) was inserted, and she was placed on mechanical ventilation and norepinephrine and dobutamine infusions. Her peak CK value was 636 IU/L at 7.5 h after admission. On day 3, the LV wall motion was hyperkinetic, and TTE revealed no apparent valvular abnormalities. Dobutamine was discontinued. However, the circulation was maintained by noradrenaline with tachycardia. CRP levels further increased to 15.02 mg/dL. Therefore, cardiogenic shock was unlikely; septic shock with acute respiratory distress syndrome was suspected. We obtained blood and sputum specimens for culture and then initiated intravenous antibiotic therapy for severe pneumonia. Vasopressin was given for septic shock because the blood pressure could not be maintained even with noradrenaline.

**Figure 3:**
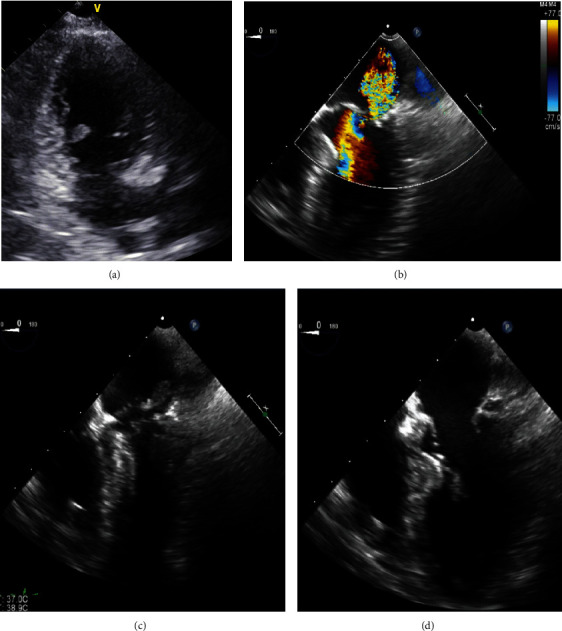
Transthoracic and transesophageal echocardiogram on day 5. (a) A fluttering mass in the left ventricle (LV), suggestive of papillary muscle rupture (white arrow) on apical two-chamber view. (b) Systolic-phase view with color doppler showing systolic jet towards the probe, indicative of mitral regurgitation. (c, d) Systolic- and diastolic-phase images of the completely ruptured posterior papillary muscle moving between the LV and left atrium (white arrow).

On day 5, the PaO2/FiO2 ratio decreased. Although there was no murmur, we reevaluated her cardiac function. Until this time, TTE could not be performed adequately because of the limited echo window and her unstable hemodynamic state. TTE was performed in the left lateral position (this was the only possible position) and showed a LV mass, suggestive of PMR (Figure 3(a)). TEE was performed, and a definitive diagnosis of acute MR and posterior PMR was confirmed (Figures 3(b)–3(d) ). We discontinued vasopressin and strongly recommended emergency mitral valve surgery; however, the patient and her family refused cardiac surgery because of her age. Subsequently, the patient was maintained with IABP. On day 7, blood cultures were negative. She died of progressive heart failure on day15.

## 3. Discussion

We encountered a case of acute MR due to PMR in a patient who presented with pulmonary edema and cardiogenic shock following AMI with small myocardial necrosis. There were some challenges encountered during diagnosis. First, although the infarct was quite small, PMR had occurred. Second, the patient had no murmur, suggesting new onset of MR. Third, the patient was finally diagnosed with PMR on TEE due to the poor quality of the images gotten on TTE. PMR can be misdiagnosed on TTE. Fourth, shock with hyperkinetic ventricular wall motion and tachycardia may be considered as one of the differential diagnoses of septic shock.

The peak CK-value generally represents the size of the MI. The peak value of CK in our patient was 636 IU/L, which was quite small. TTE showed hypokinesis in a small portion of the inferior wall. Although we expect PMR to be a complication of a large infarction, this was not the case in this patient. The posteromedial papillary muscle is supplied solely by the right coronary artery, while the anterolateral papillary muscle receives dual blood supply from the left anterior descending and circumflex arteries [1].

Our patient had no murmur, suggesting new-onset MR. It has been reported that murmurs are inaudible in almost 50% of cases of moderate to-severe acute MR, particularly those with acute ischemic MR [4]. The absence of a murmur is attributable to the rapid equalization of left atrial and ventricular pressures with decreased regurgitant volume through the mitral valve [1, 4, 5]. Therefore, the absence of murmur does not rule out acute severe MR.

TTE is often the used for the initial diagnosis of PMR; it has a sensitivity of 65%–85% [6]. However, when patients with suspected acute MR (e.g., acute heart failure with a hyperdynamic LV) cannot be diagnosed by TTE, TEE, which provides superior images of the mitral valve, can be used; moreover, the severity of regurgitation can be thoroughly assessed on TEE because of the proximity of the transducer to the mitral apparatus [1, 7].

The LV shows hyperkinetic motion due to a markedly reduced afterload. In contrast, the early phase of septic shock is hyperdynamic, that is characterized by high cardiac output and low peripheral vascular resistance [8]. Therefore, shock caused by acute MR due to PMR may be considered as one of the differential diagnoses of septic shock, as in this case.

## 4. Conclusion

Physicians should be aware of PMR associated with acute MR, following AMI with small myocardial necrosis in hemodynamically unstable patients when there is no murmur, and TTE reveals no abnormalities. TEE may be considered when poor-quality images are obtained on TTE, or diagnosis is unclear.
